# Comparative Analyses of Data Independent Acquisition Mass Spectrometric Approaches: DIA, WiSIM‐DIA, and Untargeted DIA

**DOI:** 10.1002/pmic.201700304

**Published:** 2018-01-15

**Authors:** Frank Koopmans, Jenny T. C. Ho, August B. Smit, Ka Wan Li

**Affiliations:** ^1^ Department of Molecular and Cellular Neurobiology CNCR Amsterdam Neuroscience Vrije Universiteit Amsterdam The Netherlands; ^2^ Thermo Fisher Scientific Hemel Hempstead UK

**Keywords:** data‐independent analysis, quantitative proteomics, spectral library

## Abstract

Data‐independent acquisition (DIA) is an emerging technology for quantitative proteomics. Current DIA focusses on the identification and quantitation of fragment ions that are generated from multiple peptides contained in the same selection window of several to tens of *m*/*z*. An alternative approach is WiSIM‐DIA, which combines conventional DIA with wide‐SIM (wide selected‐ion monitoring) windows to partition the precursor *m*/*z* space to produce high‐quality precursor ion chromatograms. However, WiSIM‐DIA has been underexplored; it remains unclear if it is a viable alternative to DIA. We demonstrate that WiSIM‐DIA quantified more than 24 000 unique peptides over five orders of magnitude in a single 2 h analysis of a neuronal synapse‐enriched fraction, compared to 31 000 in DIA. There is a strong correlation between abundance values of peptides quantified in both the DIA and WiSIM‐DIA datasets. Interestingly, the S/N ratio of these peptides is not correlated. We further show that peptide identification directly from DIA spectra identified >2000 proteins, which included unique peptides not found in spectral libraries generated by DDA.

LC‐MS/MS‐based quantitative proteomics is the method of choice to measure changes in global protein levels in biological samples. In the past decade, data‐dependent acquisition (DDA) has been widely used for this. In DDA, the precursors, usually the top 10–20 peptides per cycle, are sequentially selected from a full mass MS1 scan for fragmentation and acquisition in the MS/MS mode.^1^, ^2^ Recently, DDA has been optimized to reveal the comprehensive proteome of a single cell type.^3^ However, the stochastic precursor selection of DDA leads to inconsistent detection of peptides. In particular, the undersampling of medium to low abundant peptides causes high variation across replicates due to the selection of different subsets of peptides. This results in missing peptide identification, which can be substantial among replicates (>30%) and reduces the number of quantifiable proteins.^4^, ^5^

Data‐independent acquisition (DIA, also known as SWATH^6^) is a recent development in quantitative proteomics. It is mainly performed on the high‐resolution high mass accuracy mass spectrometers and has been shown to be superior to DDA^7^ by producing a higher number of quantified proteins in shorter analysis time, fewer missing values, and lower coefficients of variation (CoV) across replicates. In DIA, all peptides within a predefined wide selection window, which in the original DIA study spanned a 25 *m*/*z* range,^6^ are simultaneously fragmented. The acquisition is repeated sequentially in stepped selection windows, usually in the 400–1000 *m*/*z* range. Generally, the high number of fragments ions generated from multiple peptides contained in the same selection window complicates the analysis in a classical database search strategy. This problem is circumvented by the use of a reference spectral library, which is generated beforehand by an extensive analysis of the same/similar samples by DDA. The information of the elution time of the peptide and its fragment ions stored in the spectral library defines the identity of the peptide measured in a DIA experiment.^8^, ^9^, ^10^, ^11^ Thus, samples not present in a spectral library in principle cannot be analyzed. To circumvent this shortcoming, algorithms have been developed that create a pseudo‐DDA dataset from the DIA data (untargeted peptide identification or untargeted DIA^12^, ^13^) for subsequent search in way similar to the classical DDA strategy.

An alternative to DIA is a wide selected‐ion monitoring, DIA (WiSIM‐DIA), which is grossly underexplored. While both DIA and WiSIM‐DIA require a spectral library for peptide/protein identification, in contrast to MS2‐based DIA method, WiSIM‐DIA uses MS1 for quantitation. Previous reports on WiSIM‐DIA were performed in an Orbitrap (OT) Fusion mass spectrometer.^14^, ^15^ This method consists of three‐stepped SIM scans acquired with 240 000 resolution over a 200 *m*/*z* range that covers 400–1000 *m*/*z*. In parallel with each SIM scan, peptide fragmentation from selection windows of 12 *m*/*z* were acquired in the ion trap (IT), with acquisition repeated with 17 sequential IT MS/MS windows. In comparison, DIA used the OT for high (60 000) resolution MS1 and 17 sequential MS/MS windows in lower (15 000) resolution. So, the quality of MS1 acquisition in WiSIM‐DIA was improved compared to DIA by using stepped SIM scans and a higher resolution, while the quality of MS/MS acquisition was favorable for DIA due to the use of the OT (compared to WiSIM‐DIA using IT for MS/MS). MS/MS data acquired in the low‐resolution IT were used for identification, whereas quantitation was based on the extracted ion chromatogram of the SIM data with a 5 ppm window. Here, the spectral library could be generated with classical DDA where the MS1 full scan is acquired in the high‐resolution OT, and the fragment ions in the fast but low‐resolution IT. It is proposed that WiSIM‐DIA does not suffer from the drawback of DIA, for example, the potential interferences of the large number of fragment ions derived from coeluting peptides. However, the only application published recently reported the quantitation of about 1100 proteins by WiSIM‐DIA^[15]^, which seems to be on the lower side acquired by a modern MS. Thus, it has remained unclear whether WiSIM‐DIA is a viable alternative to DIA.

**Significance of the study**

In recent years data‐independent acquisition (DIA), the fragment ion‐centric approach, is becoming the method of choice for label‐free quantification studies. Here, we demonstrated that the precursor ion‐centric WiSIM‐DIA approach is capable of quantifying >24 000 unique peptides from a neuronal synapse‐enriched sample in 2 h analysis time, compared to 31 000 in DIA. This puts WiSIM‐DIA as a viable alternative to DIA, especially when interferences of large numbers of fragment ions derived from coeluting peptides is an issue. While data analyses of DIA and WiSIM‐DIA generally require a spectral library, recent development of untargeted DIA allows direct interrogation of raw data from a regular DIA experiment. Using this approach, we identified about 2000 proteins, from which 2000 peptides are not represented in the DDA spectral library. Therefore, the output from untargeted DIA can be added to existing spectral libraries to increase peptide identification.

In this study, we used an OT Fusion Lumos in DDA mode to generate two spectral libraries from the mouse synaptosome, a preparation enriched for proteins of the neuronal synapse,^16^ which constitutes the building block of the brain. The tryptic digest of 10 μg synaptosome proteins were fractionated offline using high pH reversed phase cartridges into eight fractions. Each fraction was subjected to DDA by two separate acquisition strategies: (1) MS1 OT with the fast but low‐resolution IT for MS/MS (HCD‐IT) and, (2) MS1 OT with the high‐resolution OT MS/MS (HCD‐OT). The data were processed using MaxQuant^17^ with 1% False Discovery Rate (FDR) at both peptide and protein level.

From the same sample, we used 1 μg for DIA with a 2 h LC gradient. Three replicates each for DIA and WiSIM‐DIA were performed. Technically, several parameters can be considered to maximize the DIA output. While a cycle scan time is usually fixed around 3–4 s to obtain six to ten measurement points of a peptide that is needed for quantitation, the width of a selection‐window, the accumulation time per selection‐window, and the whole *m*/*z* range can be varied. The original study opted for a 25 *m*/*z* selection window,^6^ which may cause peptide fragment ion interferences due to their high complexity. In another extreme, a narrow selection window of 3 *m*/*z* has been proposed as preference for more comprehensive and in‐depth view of protein profiling in a complex sample.^18^ This is compromised by a shorter acquisition time with potentially reduced sensitivity. Considering the mild protein complexity of the synaptosome fraction of about 5000 proteins contained in the spectral library, we chose the 12 *m*/*z* selection window for both DIA and WiSIM‐DIA (see also 19). The total mass range covered was 400–800 *m*/*z*, which includes the majority of the peptides (Supporting Information, Figure S1).

In addition to the classical DDA‐based spectral library, we generated a spectral library from the DIA data using the recently launched Spectronaut Pulsar software (untargeted DIA at 1% peptide and protein FDR, settings analogous to the MaxQuant DDA analysis), which yielded 17 894 unique peptide sequences in 2079 protein groups. This is less than the 27 897 and 33 673 unique peptide sequences and 4770 and 4989 protein groups represented in the IT and OT spectral libraries, respectively, within the 400–800 *m*/*z* range (Figure 1A–B, the total number of identified peptides without any *m*/*z* filters in each spectral library is shown in Supporting Information, Figures S1 and S2). Here, we compared the subset of peptides in the 400–800 *m*/*z* range to match the DDA spectral library with the acquisition settings for DIA and WiSIM‐DIA on the OT Fusion Lumos. The samples used for (untargeted) DIA were not fractionated, in contrast to the extensively fractionated samples used exclusively for DDA spectral library construction, which may account for the overall reduced number of identifications by untargeted DIA. Despite the lack of extensive fractionation, untargeted DIA contributed 2901 unique peptides to the spectral library. Interestingly, most of the peptides exclusively identified by untargeted DIA belong to protein groups that were also identified in the IT or OT libraries (or both). This suggests that the untargeted DIA unique peptides may have been lost in the first dimensional high pH reversed phase HPLC separation used for the OT and IT analyses. Alternatively, respective peptide MS/MS spectra quality could be subpar, be part of mixed chimeric spectra in shotgun MS/MS or it may due to the nature of stochastic precursor selection of DDA; while these peptides were present, they might not have been selected for MS/MS or the MS/MS could have been triggered far from the peak apex (pseudo MS/MS in untargeted DIA is generated at the apex of the peak). Either way, this argues that DIA data that are generated from routine quantitative analysis might subsequently be added to spectral libraries generated by conventional DDA to increase protein coverage.

**Figure 1:**
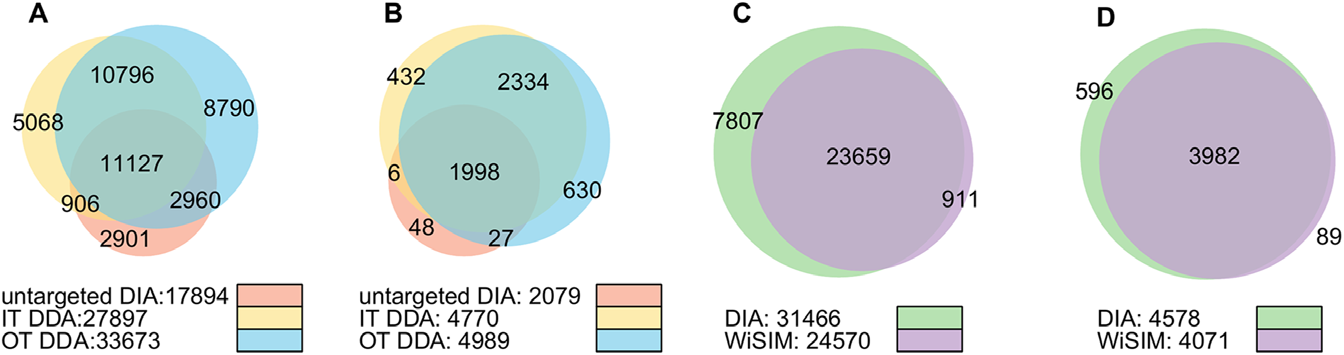
For the spectral library IT DDA, OT DDA, and untargeted DIA acquisition resulted in A) 27 897, 33 673, and 17 894 unique peptide sequences within the 400–800 *m*/*z* range and B) 4770, 4989, and 2079 protein groups, respectively. From the merged spectral library, which contains all peptides identified by either IT, OT, or untargeted DIA; C) 31 466 and 24 570 unique peptide sequences; and D) 4578 and 4071 protein groups were quantified by DIA and WiSIM‐DIA, respectively.

The performance of both DIA and WiSIM‐DIA was excellent; 31 466 and 24 570 unique peptide sequences contained in the merged spectral library were quantified at 1% peptide‐level FDR, respectively, with extensive overlap (Figure 1 C and D). These peptides map to >4000 protein groups in the merged spectral library (no protein‐level FDR was applied).

From the two spectral libraries generated using MS2 HCD‐OT or MS2 CID‐IT, we observed a slightly higher number of peptides and protein groups identified by OT (Figure 1), and a slightly higher coverage of the MS2 HCD‐OT library following DIA quantification (Figure 2). Thus, despite the slower scan rate of OT its high resolution and mass accuracy favorably affects the population of identified peptides that can be recovered in the DIA data analysis. It may also underlie the fact that both employed the same MS sector, the OT, for the measurement. The coverage of protein groups from the untargeted DIA spectral library is 100% (Figure 2), which reflects the fact that it is generated from the original DIA data. However, using the WiSIM‐DIA data, we also quantified 90 and 99% of all peptides and protein groups, respectively, from the untargeted DIA spectral library, which suggests the untargeted DIA approach tends to prioritize peptides that exhibit a clean elution profile with high S/N ratio. The spectral library coverage by WiSIM‐DIA is generally lower than that of DIA, which may underlie at least in part that current DIA algorithms are primarily using MS2 fragment intensities to identify spectral library peptides. Algorithm improvements that lead to better utilization of high‐quality MS1 signals with sub‐ppm mass error would improve the recovery rate of spectral library peptides by WiSIM‐DIA. This approach would be an extension of the previous described “accurate mass tag” strategy in which the identities of the peptides based on the LC‐FTICR MS1 measurement were validated by LC‐MS/MS analysis on a conventional IT mass spectrometer.^20^, ^21^ A similar approach has been applied to the analysis of phosphorylated human peptides,^22^ and HeLa cell proteome.^23^

**Figure 2:**
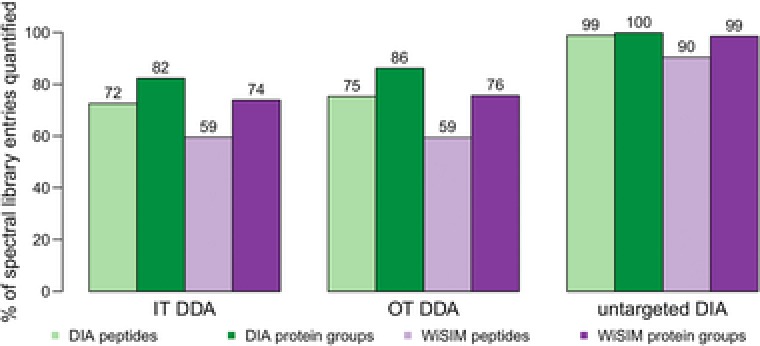
Fraction of peptide sequences and protein groups from individual spectral libraries quantified by DIA and WiSIM‐DIA. DIA quantifies on average 13% more peptides from each spectral library compared to WiSIM‐DIA. Although the number of peptides contributed to the merged spectral library is relatively low for Pulsar (Figure 1A and B), their recovery after quantification is remarkably high (note: most likely the peptides with best MS2 abundance profiles are selected for identification). Analogous figures with count data instead of fractions in Supporting Information, Figure S3.

The median coefficient of variation (CoV) for 25 788 peptides quantified in both the DIA and WiSIM‐DIA datasets was 9% within three WiSIM‐DIA technical replicates and 7% within three DIA technical replicates (Supporting Information, Figure S4). Evaluating all peptides quantified by DIA (35 123) and WiSIM‐DIA (26 899) resulted in 8 and 9% median CoV, respectively. For both comparisons, Student's *t* tests reveal the differences between DIA and WiSIM‐DIA CoV was statistically significant (*p*‐value < 10^−16^) albeit with much higher effect size for the former (Cohen's *d*: 0.29) compared to the latter (Cohen's *d*: 0.09). Correlation of the abundance values between technical replicates yielded a 0.94 *R* ^2^ and 0.93 *R* ^2^ on average for DIA and WiSIM‐DIA, respectively. The slightly reduced technical variation of DIA over WiSIM‐DIA will likely result in higher sensitivity when performing differential abundance analysis in real‐world biological applications.

The S/N ratio is a good indicator of the mass spectrometric measurement quality. In DIA mode, the S/N ratio for fragment ion intensities per precursor is better than those of the corresponding precursor measured in MS1 (Figure 3A). On the other hand, WiSIM‐DIA yields a better S/N ratio for the precursor ion measured in MS1 compared to its MS2 S/N ratio. These findings are in accordance to the experimental design that DIA is optimized for MS/MS analysis, and WiSIM‐DIA for MS1 measurement. Student's *t* tests applied to the log‐transformed WiSIM‐DIA MS1 and MS2 S/N distributions confirmed statistical significance of this comparison (*p*‐value < 10^−16^) with a medium‐large effect size (Cohen's *d*: 0.67). Analogously, the overall DIA MS2 S/N distribution was significantly lower (*p*‐value < 10^−16^) than its WiSIM‐DIA MS1 counterpart, but with a relatively small effect size (Cohen's *d*: 0.34). In addition to comparing the distributions shown in Figure 3A, Student's *t* tests on log2 DIA MS2 and WiSIM‐DIA MS1 S/N values for all individual peptides using the triplicate DIA and WiSIM‐DIA measurements resulted in 4155 (out of 25 780) significantly different peptides at FDR‐adjusted *p*‐value ≤ 0.01. Of these, 1010 and 3145 peptides showed improved S/N in WiSIM‐DIA MS1 and DIA MS2, respectively. We found a strong correlation (0.792 *R* ^2^) between the abundance values of peptides quantified in both the DIA and WiSIM‐DIA datasets, as expected (Figure 3B). Interestingly, the S/N ratio of these peptides was not correlated (0.171 *R* ^2^) between DIA and WiSIM‐DIA (as compared to 0.80 and 0.59 average *R* ^2^ between DIA and WiSIM‐DIA replicates, Supporting Information, Figure S5). The lack of correlation in S/N might be explained by the stepped SIM scans in WiSIM‐DIA that clean up the spectra of many peptides, but might reduce the signal for already low abundant peptides (Figure 3C). The use of different modes of quantification for WiSIM‐DIA (MS1) and DIA (MS2) taken together with their overall similar S/N distributions shown in Figure 3A could give rise to subpopulations of peptides that are quantified with higher signal quality in either WiSIM‐DIA or DIA, indicating mutually exclusive benefits. Alternatively, observed differences in peptide subsets could arise by chance. Future research could further investigate this hypothesis using extensive datasets that allow for cross‐validation of peptides with stark differences in S/N between WiSIM‐DIA and DIA.

**Figure 3:**
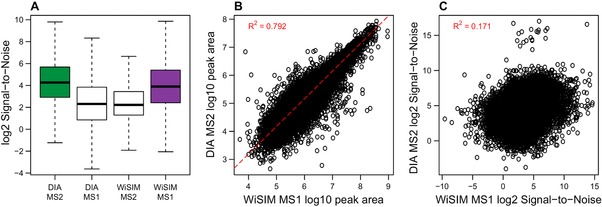
Quality of precursor quantification compared between DIA (MS2) and WiSIM‐DIA (MS1). A) The S/N ratio is high for both DIA MS2 and WiSIM‐DIA MS1. Analogously, the secondary mode of quantification (MS1 for DIA, MS2 for WiSIM‐DIA) is more noisy. B) The abundance values of 25 778 precursors quantified by both DIA and WiSIM‐DIA are correlated (0.792 *R* ^2^), as expected. The dashed red line shows linear regression. C) Interestingly, the S/N for these peptides is not correlated (0.171 *R* ^2^). A subpopulation of precursors is quantified with higher signal quality by DIA MS2 than WiSIM‐DIA MS1, and vice versa.

We conclude that DIA and WiSIM‐DIA can quantify more than 31 000 and 24 000 unique peptides (at 1% peptide‐level FDR), respectively, over five orders of magnitude in a single 2 h analysis with nearly no missing values (0.08 and 0.004% missing peptide values between three technical replicates, respectively). The number of peptides from the spectral libraries recovered by WiSIM‐DIA will be improved when its high‐quality MS1 signal is better taken advantage of by future improvements of analysis software (e.g., by relying on accurate retention time and low precursor mass error for matching precursor peaks to the library in absence of high‐quality fragment spectra^20^, ^21^). The untargeted DIA spectral library generated from the triplicate 2 h DIA analysis yields nearly 50% of the peptides/proteins contained in the spectral library generated from the 8 × 2 h analysis of the deep MS sequencing of the sample, as well as unique peptides. We anticipate that a narrow selection window of a few *m*/*z* (SWATH‐ID of 3 *m*/*z* 18) analyzed in a fast machine such as Q‐Exactive HF‐X with 40 Hz will generate a untargeted DIA library that might be of competitive quality with the classically generated spectral library, however, with much reduced analysis time, which is also a better match to the subsequent DIA analysis using similar LC‐MS/MS parameters.

**Abbreviations**
DDA: data‐dependent acquisition
DIA: data‐independent acquisition
HCD: Higher‐energy collisional dissociation
IT: Ion Trap
OT: Orbitrap
SIM: selected‐ion monitoring
WiSIM: wide selected‐ion monitoring

## Conflict of Interest

The authors have declared no conflict of interest.

## Supporting information

Supporting information
