# Supplementary material for: Comparative Analyses of Data Independent Acquisition Mass Spectrometric Approaches: DIA, WiSIM‐DIA, and Untargeted DIA
Source: Proteomics. 2018 Jan 15;18(1):1700304. doi: 10.1002/pmic.201700304 (PMC5817406; doi:10.1002/pmic.201700304)
Supplement: Supplementary file 1 — Supporting information [file PMIC-18-na-s001.docx]

Supplementary Figures

**Comparative analyses of data independent acquisition mass spectrometric approaches: DIA, WiSIM-DIA and untargeted DIA**

Frank Koopmans^1^, Jenny Ho^2^, August B. Smit^1^ and Ka Wan Li^1^

^1^Department of Molecular and Cellular Neurobiology, Center for Neurogenomics and Cognitive Research, Amsterdam Neuroscience, VU Amsterdam, Amsterdam, The Netherlands

^2^Thermo Fisher Scientific, Hemel Hempstead, UK

Corresponding author: Frank Koopmans, Department of Molecular and Cellular Neurobiology, Vrije Universiteit Amsterdam, De Boelelaan 1085, 1081HV, Amsterdam, The Netherlands

E-mail: [frank.koopmans@vu.nl](mailto:frank.koopmans@vu.nl)


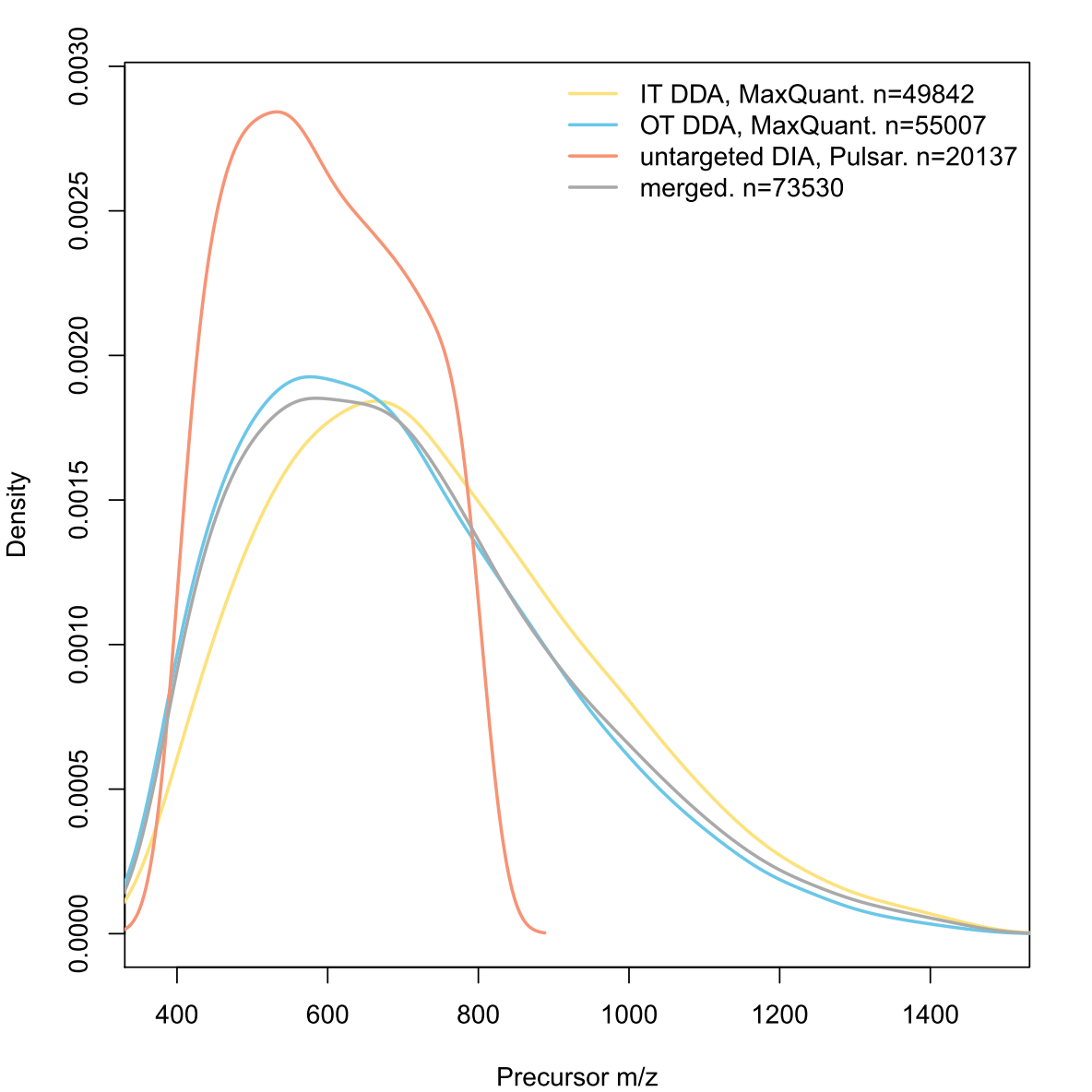


Supplementary Figure S1. Precursor m/z distribution for all peptides from each spectral library.


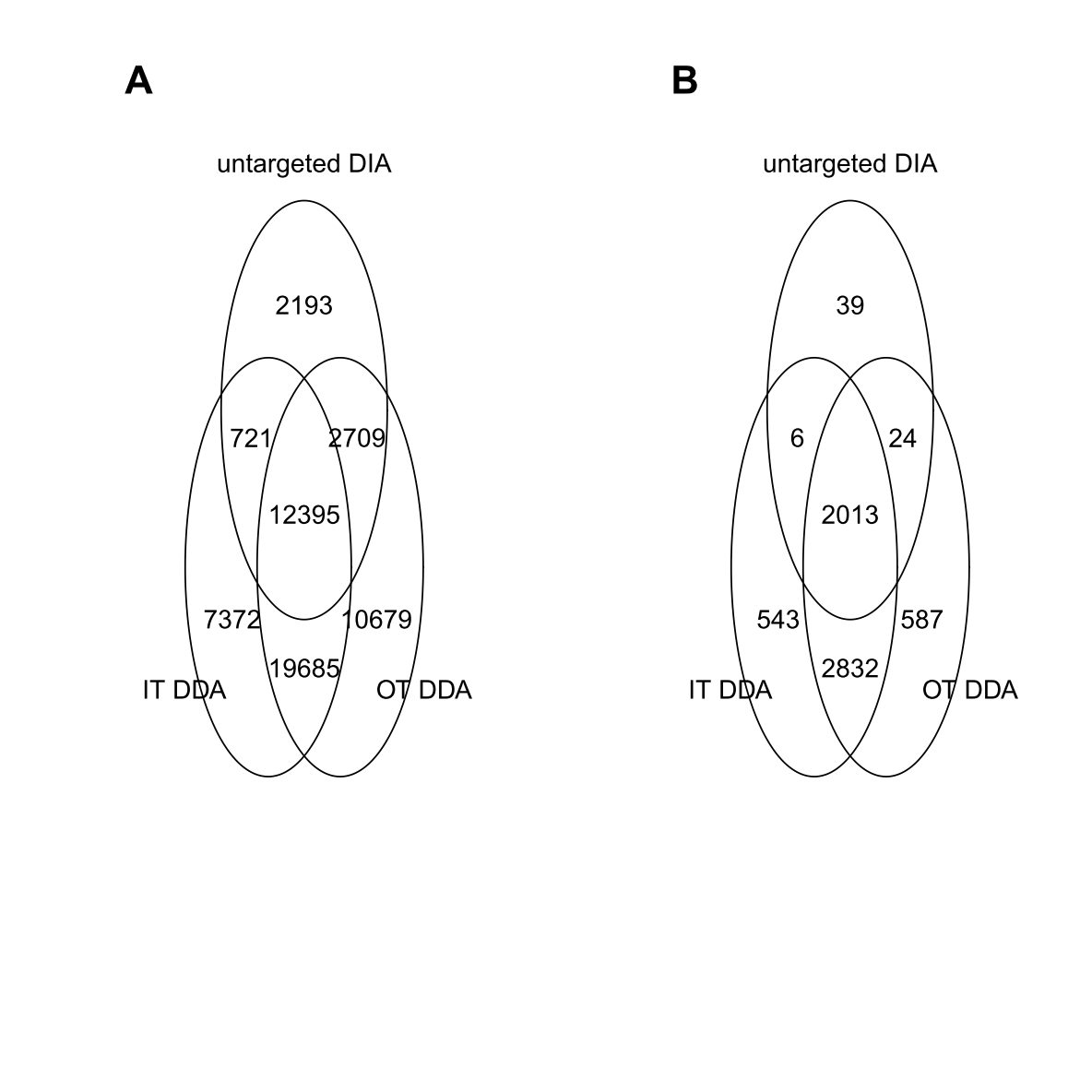


Supplementary Figure S2. All identified A) unique peptide sequences and B) protein groups in each spectral library compared in a Venn diagram. Related to Figure 1A-B, but here we do not filter for precursors in the 400-800 m/z range.


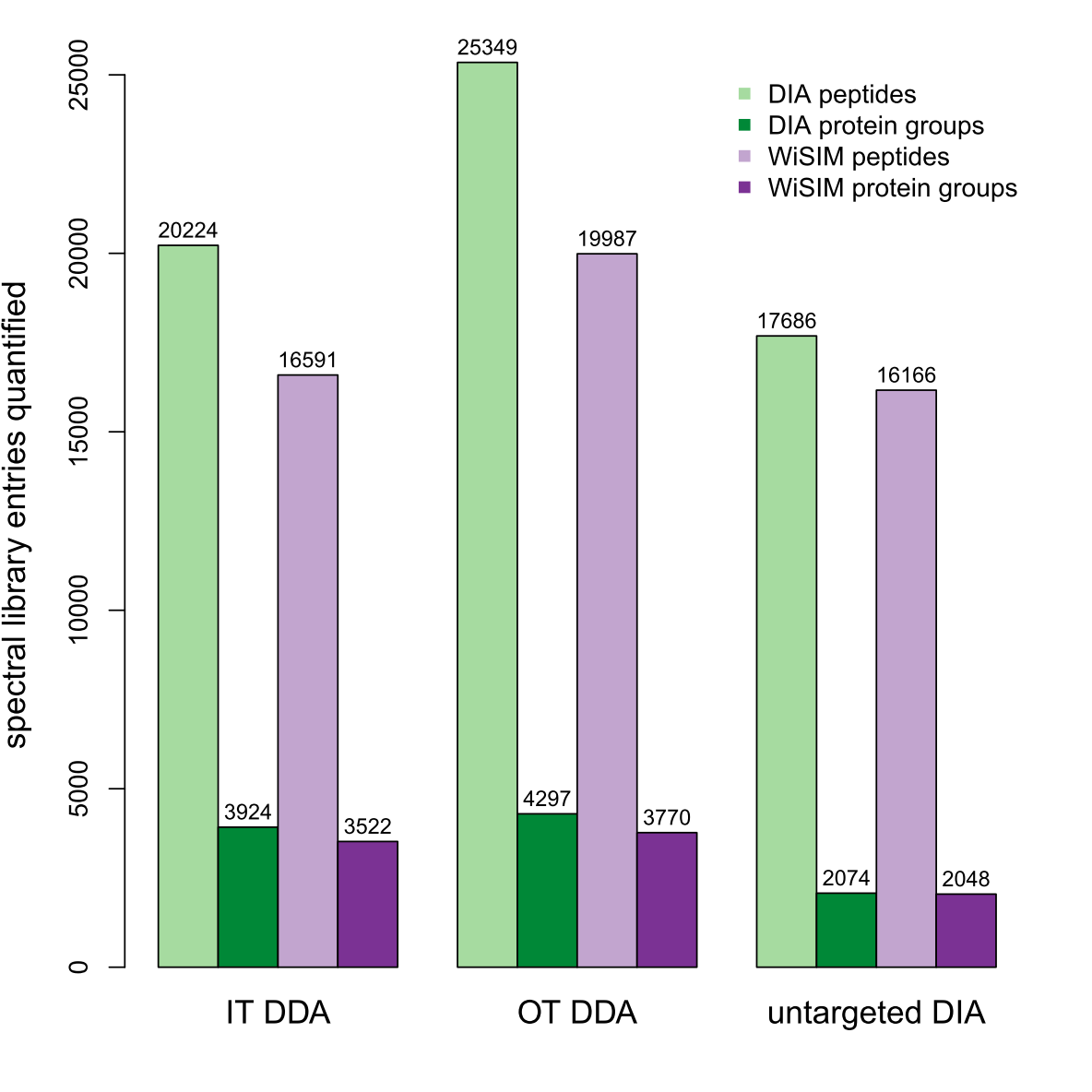


Supplementary Figure S3. Peptide sequences and protein groups from individual spectral libraries quantified by DIA and WiSIM-DIA. Analogous to Figure 2.


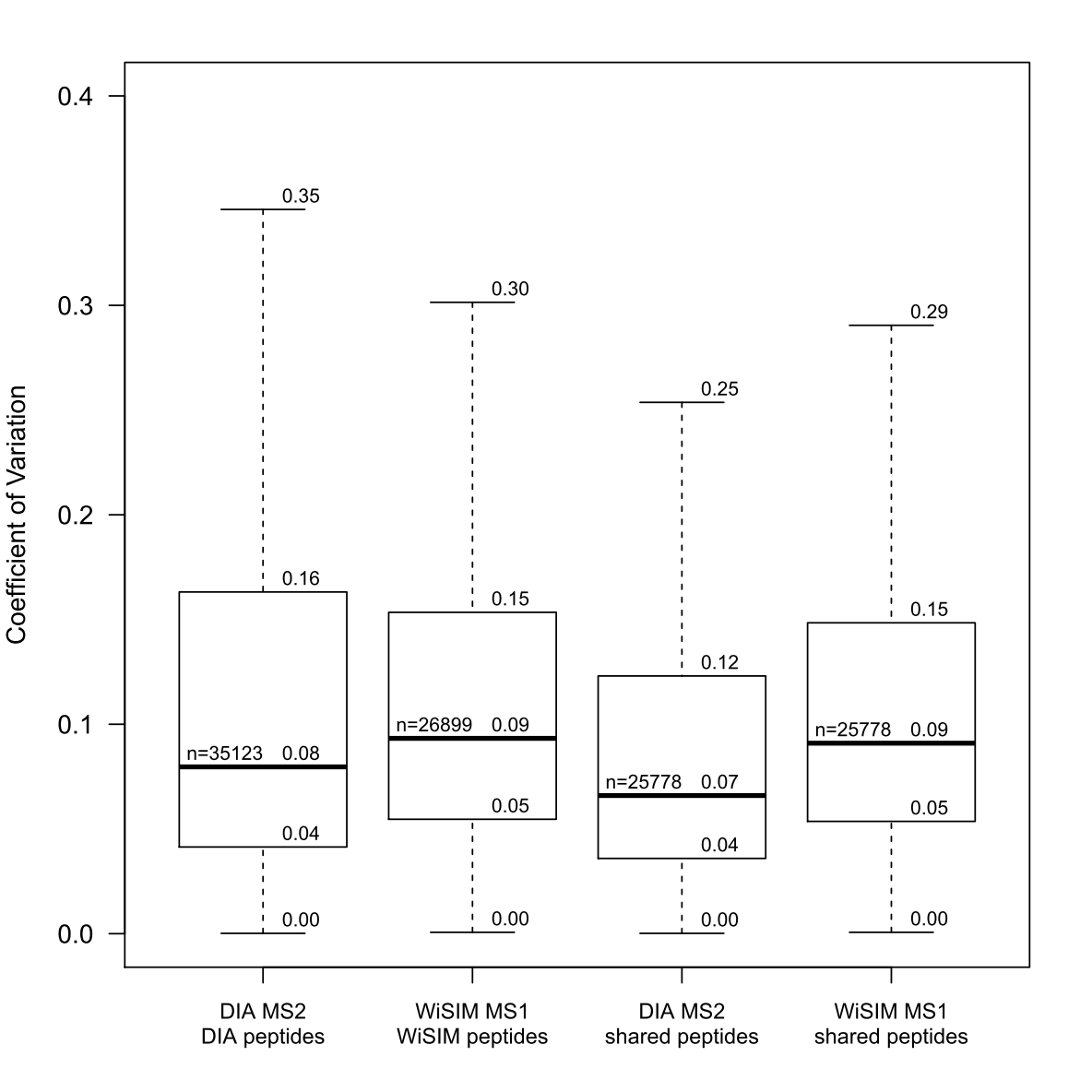


Supplementary Figure S4. Coefficient of Variation (CoV) for triplicate measurements of DIA-MS2 and WiSIM-MS1. Different sets of peptides were used for the evaluation of technical reproducibility; *DIA peptides* refers to the set of peptides from the merged spectral library that were recovered in at least 2/3 DIA technical replicates with Q-value ≤ 0.01, analogously *WiSIM peptides* refers to peptides recovered in 2/3 WiSIM technical replicates with Q-value ≤ 0.01 and *shared peptides* was defined as the union of both sets (those peptides confidently recovered with DIA and WiSIM). The number of peptides that was used for the CoV computation, *n*, in each set is shown within the boxplot.


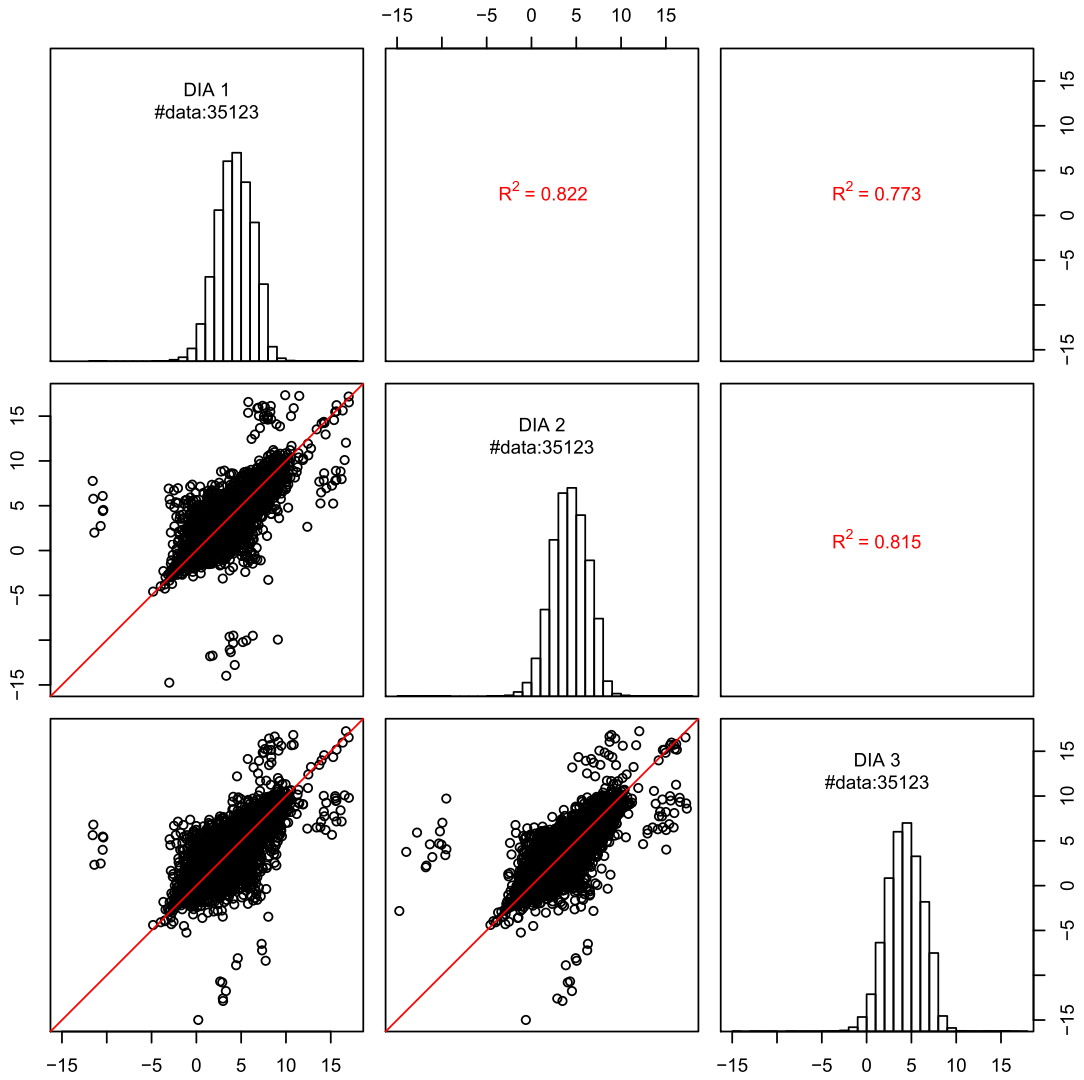
**A**


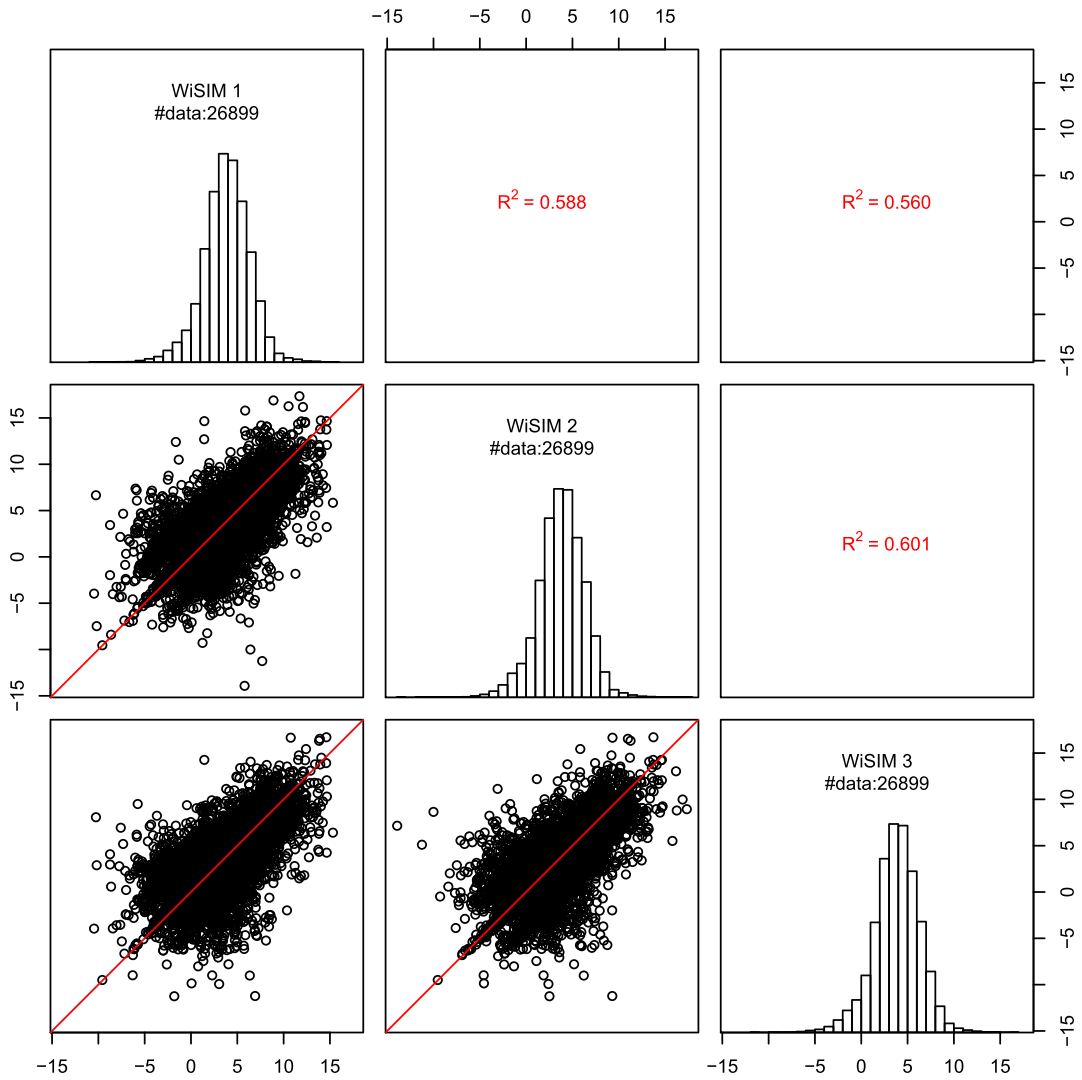


**B**

Supplementary Figure S5. Pairwise scatterplots of the log2 signal to noise ratio (S/N) between replicates of A) DIA MS2 and B) WiSIM-DIA MS1. For both methods, the subset of peptides with Q-value ≤ 0.01 in at least 2/3 technical replicates was used. The distribution of values for each sample is shown on the diagonal, together with the sample name and the number of datapoints.

**A**

**
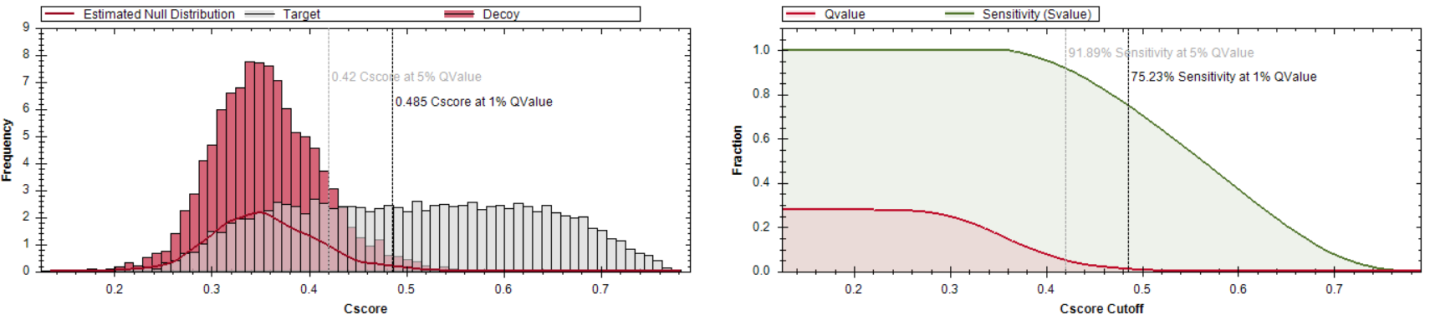
**

**B**

**
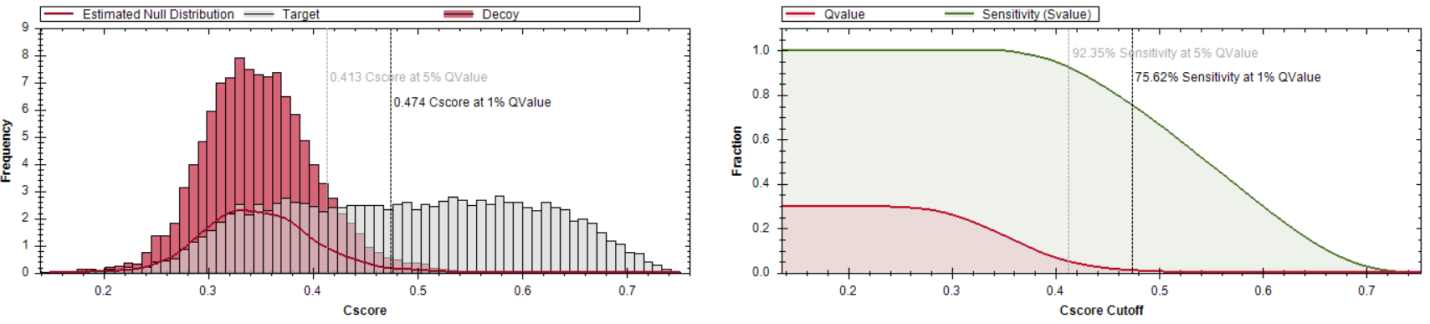
**

**C**

**
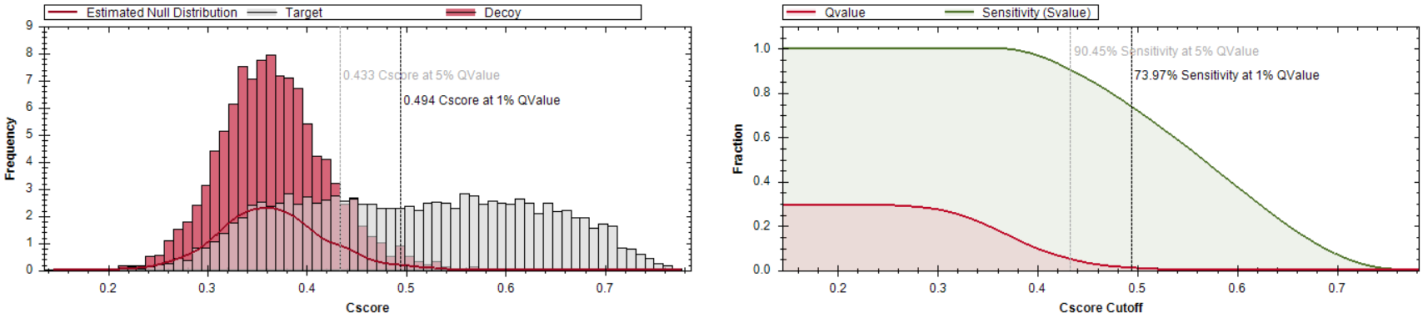
**

**D**

**
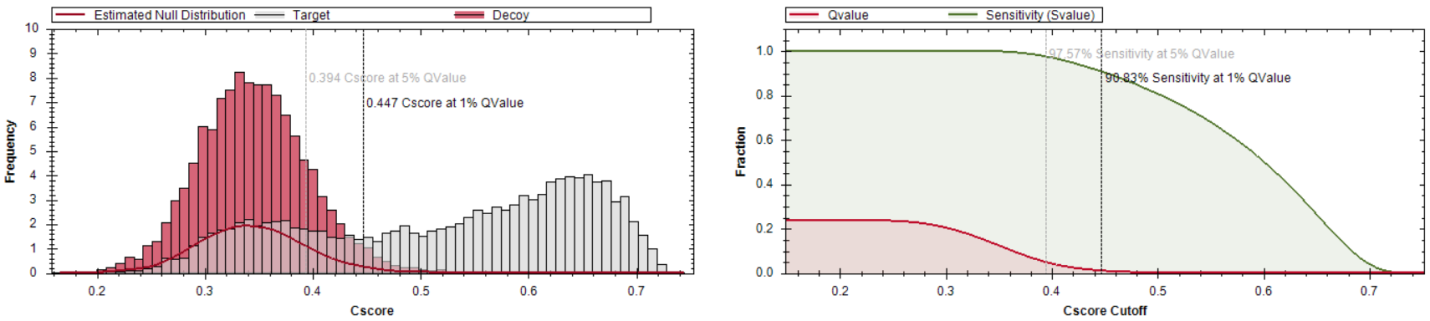
**

**E**

**
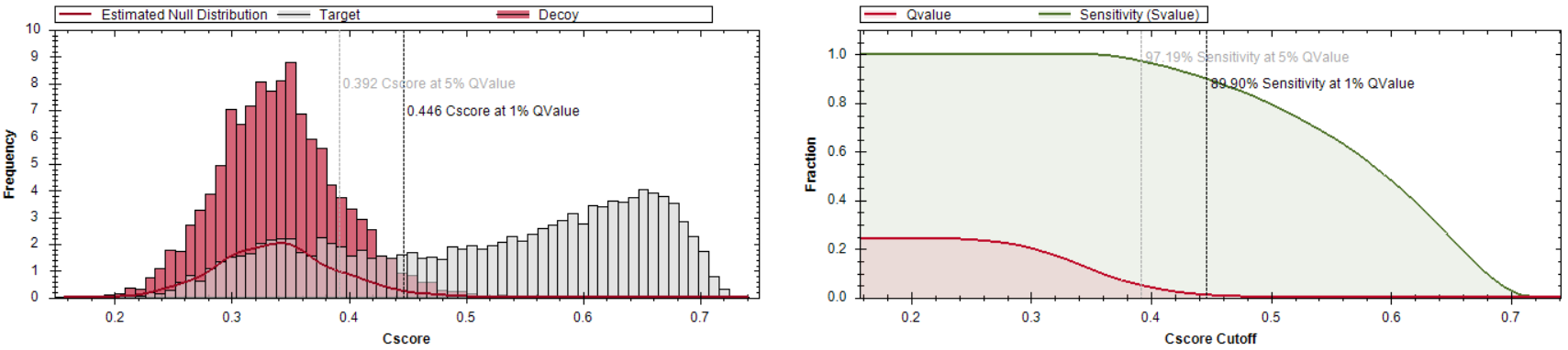
**

**F**


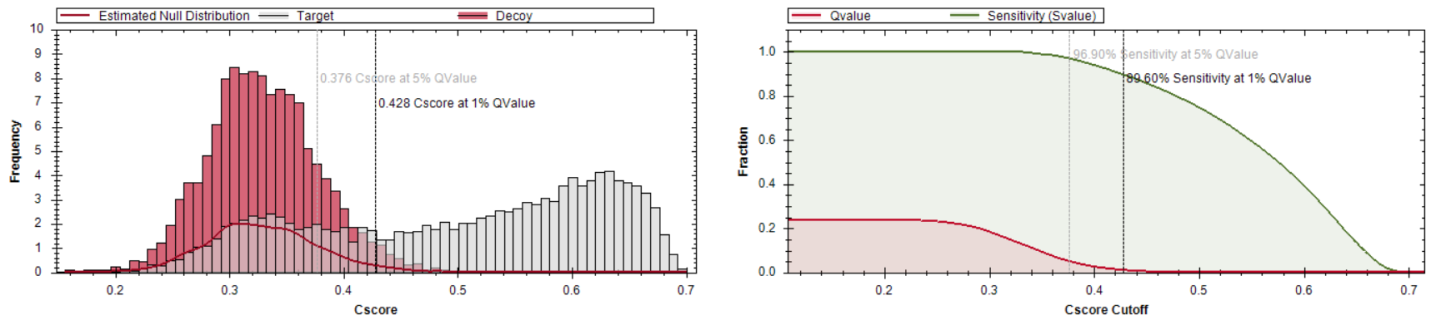


Supplementary Figure S6. Spectral library matching score distributions from quantitative analysis in Spectronaut for (A-C) WiSIM samples 1-3 and (D-F) DIA samples 1-3.
